# Paracrine Activation of STAT3 Drives GM-CSF Expression in Breast Carcinoma Cells, Generating a Symbiotic Signaling Network with Breast Carcinoma-Associated Fibroblasts

**DOI:** 10.3390/cancers16162910

**Published:** 2024-08-22

**Authors:** Kingsley O. Osuala, Anita Chalasani, Neha Aggarwal, Kyungmin Ji, Kamiar Moin

**Affiliations:** 1 Department of Pharmacology, Wayne State University School of Medicine, 540 East Canfield, Detroit, MI 48201, USA; ag6370@wayne.edu (A.C.); kji@med.wayne.edu (K.J.); 2 Twelve Biosciences Research & Development, Kalamazoo, MI 49009, USA; 3 Department of Physiology, Wayne State University School of Medicine, 540 East Canfield, Detroit, MI 48201, USA; nehaaggarwal.bio@gmail.com; 4 Department of Neurology, Henry Ford Health, Detroit, MI 48202, USA

**Keywords:** breast carcinoma, breast carcinoma-associated fibroblasts, GM-CSF, STAT3, paracrine signaling

## Abstract

**Simple Summary:**

Granulocyte-macrophage colony-stimulating factor (GM-CSF) is a monomeric glycoprotein most commonly associated with myeloid cell differentiation and the stimulation of inflammatory processes. Several studies have shown that cancer cells express GM-CSF; however, the mechanism and effect of this expression has not been fully resolved. Some studies suggest the levels of GM-CSF expression and secretion determine its role in tumor suppression or growth. We believe and begin to outline in the current study, that breast cancer cell expression and secretion of GM-CSF is an operative pro-survival tool utilized to induce fibroblasts, macrophages, and other myeloid-derived cells to produce pro-inflammatory cytokines and growth factors such as interleukins 6 and 8. These pro-survival factors drive tumor progression. Understanding these paracrine signaling networks in the tumor microenvironment will facilitate the development of targeted and efficient therapies to halt tumor progression.

**Abstract:**

This study evaluated the paracrine signaling between breast carcinoma-associated fibroblasts (CAFs) and breast cancer (BCa) cells. Resolving cell–cell communication in the BCa tumor microenvironment (TME) will aid the development of new therapeutics. Here, we utilized our patented TAME (tissue architecture and microenvironment engineering) 3D culture microphysiological system, which is a suitable pathomimetic avatar for the study of the BCa TME. We cultured in 3D BCa cells and CAFs either alone or together in cocultures and found that when cocultured, CAFs enhanced the invasive characteristics of tumor cells, as shown by increased proliferation and spread of tumor cells into the surrounding matrix. Secretome analysis from 3D cultures revealed a relatively high secretion of IL-6 by CAFs. A marked increase in the secretion of granulocyte macrophage-colony stimulating factor (GM-CSF) when carcinoma cells and CAFs were in coculture was also observed. We theorized that the CAF-secreted IL-6 functions in a paracrine manner to induce GM-CSF expression and secretion from carcinoma cells. This was confirmed by evaluating the activation of STAT3 and gene expression of GM-CSF in carcinoma cells exposed to CAF-conditioned media (CAF-CM). In addition, the treatment of CAFs with BCa cell-CM yielded a brief upregulation of *GM-CSF* followed by a marked decrease, indicating a tightly regulated control of *GM-CSF* in CAFs. Secretion of IL-6 from CAFs drives the activation of STAT3 in BCa cells, which in turn drives the expression and secretion of GM-CSF. As a result, CAFs exposed to BCa cell-secreted GM-CSF upregulate inflammation-associated genes such as *IL-6*, *IL-6R* and *IL-8*, thereby forming a positive feedback loop. We propose that the tight regulation of *GM-CSF* in CAFs may be a novel regulatory pathway to target for disrupting the CAF:BCa cell symbiotic relationship. These data provide yet another piece of the cell–cell communication network governing the BCa TME.

## 1. Introduction

### 1.1. Breast Cancer

In the latest global cancer statistics data, breast cancer is listed as the most common cancer in women worldwide [1], with more than two million new breast cancer cases are projected for 2024 [2]. In order to gain a better understanding of breast cancer development and its progression from indolent disease to metastatic disease, we need to better understand how cells communicate with one another and how they interact with other elements in their microenvironment.

Cancer cells represent a fraction of the constituents of the breast tumor microenvironment (TME). Several other cell types including macrophages, fibroblasts, adipocytes, and T-lymphocytes share the TME with tumor cells. In addition to the various cell types, the TME contains an interlaced network of structural components including laminins [3,4], collagen [5], and integrins [6,7], which influence cell behavior and store cell signaling factors such as cytokines and growth factors [8,9]. Many of these components play a direct or indirect role in tumor progression. Other aspects to consider within the breast TME are the biophysical properties of the ECM, i.e., biomechanical forces, collagen density, and elasticity [10,11]. Paracrine signaling via cytokines and growth factors is a component of tumor progression that is little understood. This study evaluates how paracrine signaling influences the breast carcinoma cell behavior in the 3D TME.

### 1.2. Cytokines/Growth Factors as Therapeutic Targets

To date, several breast cancer therapeutics target either cytokines or growth factors. For example, Tamoxifen, a drug commonly used to treat estrogen receptor (ER)-positive breast cancers, competitively binds to the ER, preventing estrogen from binding and thereby diminishing estrogen-induced tumor progression [12]. Another commonly prescribed therapy is Trastuzumab, a monoclonal antibody against the human epidermal growth factor receptor 2 (HER-2) [13]. Emerging studies of a monoclonal antibody targeting the cytokine IL-6 show promise for the treatment of some breast cancers [14,15]. There are many other cytokines and growth factor-based therapeutics in the research pipeline including tumor necrosis factor alpha and transforming growth factor beta [16,17,18,19]. Another target is the granulocyte macrophage colony-stimulating factor (GM-CSF), which is known to be a powerful myeloid immunostimulant [20]. A recombinant GM-CSF is currently used clinically to shorten the time to neutrophil recovery following chemotherapy in patients 55 years and older with acute myeloid-leukemia-(AML) [21].

In this study, we will examine the role of GM-CSF signaling between human breast tumor cells and human breast fibroblasts in the context of a 3D microenvironment. Endogenous GM-CSF is primarily secreted from activated T and B cells, macrophages, and other cells involved in immune response [22,23]. Signaling occurs through binding of GM-CSF to its receptor (GM-CSFR), which is expressed at the cell surface of myeloid, endothelial cells, and fibroblasts [24,25]. GM-CSF is also secreted from several tumors including lung, brain, bladder, colorectal, and metastatic breast [26,27]. However, little data are available which assess the expression and secretion of GM-CSF from early stage and pre-metastatic human BCa cells. Recombinant GM-CSF has been shown to inhibit BCa cell growth in mouse models [28,29], and when overexpressed was able to sensitize BCa cells to chemotherapeutics [30] suggesting that GM-CSF plays an anti-cancer role. Conversely, others have found that GM-CSF is a mediator of macrophage infiltration [31], which is a poor prognostic factor for breast cancer patient survival [32].

### 1.3. Carcinoma-Activated Fibroblasts (CAFs) and GM-CSF

CAFs are a subset of fibroblasts present in the TME that express a host of proinflammatory factors and matrix degradation proteases [33]. According to the available literature, CAFs express both GM-CSF and GM-CSFR [34,35]. Many studies have identified CAFs as constituents of the tumor microenvironment including tumors of the head and neck [36], prostate [37], lung [38] and ovaries [39]. Our group along with others have shown that CAFs contribute to breast cancer progression by increasing matrix degradation and guiding tumor cells through the surrounding ECM and away from the primary tumor structure [40,41,42]. There is also evidence that CAFs are further apportioned into subsets characterized by genetic, phenotypic, and spatial positioning relative to tumor cells. Wu et al. describe four distinct states in which CAFs exist in human triple negative BCa: myofibroblast-like or inflammatory-like CAFs and differentiated or immature-perivascular-like cells [43]. However, it is unknown whether the protein secretion profile varies among these four subtypes. Characterizing the secretion profile of CAFs in the TME may aid in understanding the paracrine signaling network(s) involved and their effects on tumor progression.

In the current study, we evaluated paracrine GM-CSF signaling between BCa cells (preinvasive and invasive) and breast CAFs in the context of a 3D culture system. Our in vitro 3D tissue architecture and microenvironment engineering or TAME culture model (Supplemental Figure S1) allows for high-content data generation, including imaging of the spatiotemporal landscape of cell-to-cell interactions, evaluation of extracellular matrix remodeling, and characterization of tumor architectural changes in response to dose-dependent drug therapies. We utilized our novel TAME 3D culture model to better understand the mechanisms by which carcinoma cells and CAFs communicate and interact. We show that breast carcinoma cells and CAFs sustain a cooperative relationship through a STAT3-driven positive feedback loop that stimulates a pro-tumorigenic phenotype in breast carcinoma cells in a 3D culture system.

## 2. Materials and Methods

### 2.1. Cell Lines

MCF10.DCIS human breast carcinoma cells are a preinvasive variant of the MCF-10A cell line [44]. HCC70 human breast carcinoma cells are a poorly differentiated cell line derived from an invasive primary ductal carcinoma. CAF40T human breast CAFs were isolated from a biopsy of an invasive ductal carcinoma by Dr. Simon W. Hayward (NorthShore University HealthSystem Research Institute) and were immortalized in the laboratory of Dr. Bonnie Sloane (Wayne State University) to develop the CAF40TKi cell line (hereafter called CAFs). All cell lines were authenticated by the Karmanos Cancer Institute’s Biobanking and Correlative Sciences Core at Wayne State University. Additionally, cell lines were routinely tested by RT-PCR to ensure that they were free of mycoplasma contamination.

### 2.2. Cell Culture

Human MCF10.DCIS and HCC70 breast carcinoma cell lines were maintained as previously described [15]. All cells grown using our TAME 3D model were set up and maintained as previously described (Supplemental Figure S1) [45]. Briefly, cell culture dishes were coated with 100% Cultrex^TM^ (Reduced Growth Factor Basement Membrane Extract, R&D systems, Minneapolis, MN, USA). The Cultrex^TM^ was allowed to solidify before cells were seeded on top. Cells adhered to the matrix within 45 min. At this point, an overlay of 2% Cultrex^TM^ (*v*/*v*) in phenol red-free Dulbecco’s Modified Eagle Medium [Nutrient Mixture F12 (DMEM/F12)] containing 2.5% fetal bovine serum (FBS) was added on top of the adherent cells.

Human breast CAFs were maintained in MCDB 131 medium without glutamine and supplemented with 10% FBS *v*/*v*, 1% MEM non-essential amino acid solution (100X), 1% insulin/transferrin/selenium/sodium pyruvate solution also known as ITS-A *v*/*v*, 12% AminoMAX^TM^ C-100 Basal Medium *v*/*v*, 5% AminoMAX^TM^ C-100 Supplement *v*/*v*, 1% pen/strep, and 2.5 mM L-glutamine from ThermoFisher Scientific (Waltham, MA, USA). Control Medium: MCDB 131 and supplements without FBS.

For cocultures, CAFs were seeded first on Cultrex^TM^ and allowed to adhere, followed by breast carcinoma cells being seeded on top of CAFs at a ratio of 5 breast carcinoma cells to 1 CAFs approximately 45 min later. A 2% mixture of CultrexTM in DMEM/F12 overlay was added after carcinoma cells adhered. All cultures were kept in a 37 °C incubator with 5% CO_2_.

Drug treatments: Niclosamide was used as a STAT3 inhibitor and was purchased from Caymen Chemical (Ann Arbor, MI, USA). Stattic, a small-molecule STAT3 inhibitor, was purchased from Sigma (St. Louis, MO, USA). Stattic and niclosamide were prepared in DMEM/F12 immediately prior to use. Recombinant IL-6 (cat#, 206-IL-010, R&D Systems, Minneapolis, MN, USA) was prepared from diluted frozen stocks. Recombinant GM-CSF (cat#, AF-300-03) was purchased from Peprotech (Rocky Hill, NJ, USA). Final drug concentrations were as follows: Niclosamide 250 nM, stattic 30 μM, recombinant IL-6 100 ng/mL, and recombinant GM-CSF 1 ng/mL.

Conditioned media (CM): Breast CAFs or MCF10.DCIS cells were cultured using the TAME 3D model for 8 days. On day 8, fresh media were added and then collected after 24 h exposure to the corresponding cell type. The collected CM were flash frozen in liquid nitrogen until use.

### 2.3. Image Acquisition and Quantitative Analysis

Differential interference contrast (DIC) or fluorescent images of 16 contiguous fields of 3D structures from live-cell imaging were acquired using either a Zeiss LSM 510 or LSM 780 upright confocal microscope (Zeiss, White Plains, NY, USA) at Wayne State University’s Microscopy, Imaging and Cytometry Resources Core facility (MICR). Images were reconstructed in 3D using Volocity 6.3 software (PerkinElmer, Waltham, MA, USA). Total volumes of 3D structures were quantified using Volocity 6.3 software as previously described [46]. Red fluorescent protein (RFP)-labeled cells were used for confocal microscopy. Area measurements were performed in ImageJ 1.54e. Tumor spheroidal structures were selected randomly (*n* = 10), and the area was obtained by outlining the circumference and calculating the enclosed area.

### 2.4. Western Blot

Monolayer cell cultures were grown to ~70% confluency in 100 mm dishes and CAF-CM (with or without STAT3 inhibitors niclosamide or static) was added. After a 30 min incubation period, cells were washed twice with PBS. PBS was decanted followed by the addition of 1 mL of chilled RIPA lysis buffer containing protease/phosphatase inhibitors (Cell Signaling Technology, Danvers, MA, USA). Cells were harvested using a cell scraper and transferred to conical tubes on ice followed by a 30 s sonication. The lysates were centrifuged at 14,000 rpm for 10 min, and the supernatant collected for immunoblotting. Western blot experiments were performed using 2D cultures due to excess 3D matrix protein carryover, which produced smears in electrophoresis gels.

### 2.5. ELISA and Inflammation Array

CM and cell lysates were obtained from TAME 3D cultures for analysis of cytokines and STAT3 phosphorylation. STAT3 ELISA kits (RAB0444, RAB0447) were purchased from Sigma. Human Inflammation Arrays (ab134003) were purchased from Abcam (Cambridge, MA, USA). Experiments were performed according to the manufacturer’s recommended protocols.

### 2.6. Antibodies

Primary Stat3 [STAT3 (D3Z2G), phospho-Stat3-(D3A7), phospho-Stat3-(6E4)] and beta-actin (#4967) antibodies were purchased from Cell Signaling Technology.

### 2.7. DNA Extraction and Quantification

Genomic DNA was extracted from 3D monoculture and coculture samples at 1-day or 8-day time-points. Extraction was performed using the OmniPrep^TM^ DNA isolation kit (G-Biosciences, St. Louis, MO, USA). Briefly, cells and Cultrex^TM^ were scraped from culture dishes at either 1-day or 8-day timepoints after seeding. The entire lysate was mixed 1:1 (*v*/*v*) with a genomic lysis buffer and then incubated with proteinase K at 55 °C for one hour. After the incubation period DNA extraction was performed, as described in the manufacturer’s protocol. The DNA concentration was determined using a NanoDropTM 2000 spectrophotometer (ThermoFisher Scientific). Cell proliferation was assessed using the Click-it EDU system (ThermoFisher Scientific). Fluorescent imaging of the EDU (5-ethynyl-2′-deoxyuridine) probe was performed on cocultures at day 5 of an 8-day culture period.

### 2.8. Real-Time PCR

RNA from TAME 3D cultures was extracted using TRIzol^TM^ reagent (ThermoFisher Scientific). Cells were washed 3 times with PBS then harvested using a cell scraper. The cells were transferred to tubes containing ice-cold PBS/EDTA (Sigma). The tubes were placed on ice and agitated on a rocker for 1 h at 4 °C. The cells were spun down at 1500 rpm for 5 min and the supernatant was discarded. This was followed by the addition of TRIzol^TM^ reagent and RNA extracted according to the manufacturer’s protocol. All RNA was DNAse-treated and reverse-transcribed using the High-Capacity cDNA Reverse Transcription Kit (ThermoFisher Scientific). Gene expression levels were determined using Taqman assays (ThermoFisher Scientific).

### 2.9. Statistics

All statistics were performed using GraphPad’s Prism software, release versions 7 and 10. Comparative analyses were all performed using either Student’s *t*-test or one-way ANOVA.

## 3. Results

### 3.1. Three-Dimensional Coculture of BCa Cells and CAFs Enhanced Carcinoma Cell Proliferation

**Figure 1:**
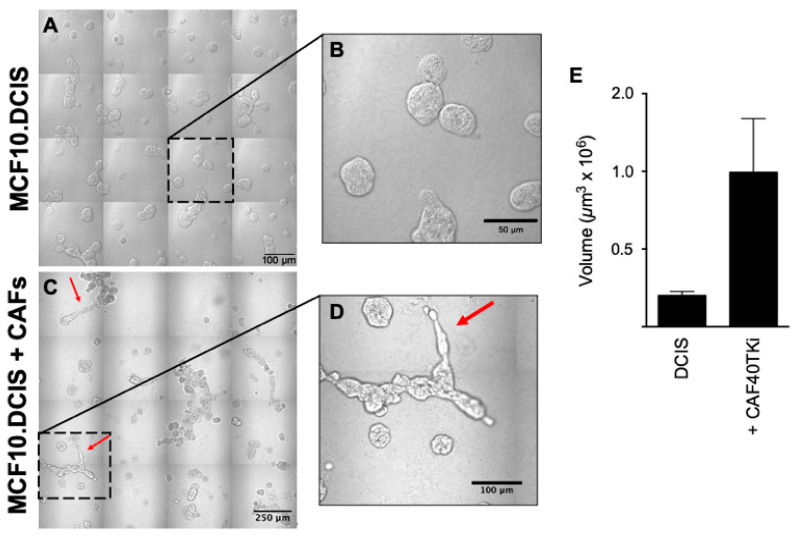
Coculture of MCF10.DCIS BCa cells and CAF40TKi CAFs resulted in BCa cell proliferation and increased tumor volume. Cells were grown for a period of 8 days in TAME 3D culture in the absence or presence of CAFs. (**A**) Image of 16 contiguous DIC fields of MCF10.DCIS cells alone or (**C**) in coculture with CAFs. Note in the high-magnification panels (**B**,**D**) the absence and consequent appearance of protrusions extending from multicellular structures (red arrows). (**E**) Quantification shows contrast in the volume of MCF10.DCIS BCa structures ± CAFs (*p*-value = 0.15, *n* = 3). Graphical data are expressed as mean ± standard deviation using Student’s *t*-test. Scale bars, (**A**) 100 microns, (**B**) 50 microns, (**C**) 250 microns, and (**D**) 100 microns.

**Figure 2:**
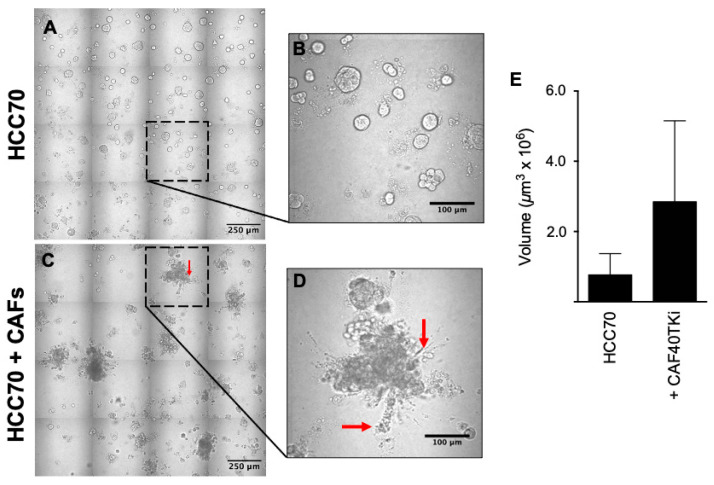
Coculture of HCC70 BCa cells and CAF40TKi CAFs resulted in BCa cell proliferation and increased tumor volume. HCC70 cells were grown for a period of 8 days in TAME 3D culture in the absence or presence of CAFs. (**A**) Image of 16 contiguous DIC fields of HCC70 cells alone or (**C**) in coculture with CAFs. Note in the high-magnification panels (**B**,**D**) the absence and consequent appearance of protrusions extending from multicellular structures (red arrows). (**E**) Quantitative comparison shows contrast in the volume of HCC70 BCa structures ± CAFs (*p*-value = 0.13, *n* = 3). Graphical data are expressed as mean ± standard deviation using Student’s *t*-test. Scale bars: (**A**,**C**) 250 microns; (**B**,**D**) 100 microns.

We compared the malignant phenotypes of two human breast carcinoma (BCa) cell lines, preinvasive MCF10.DCIS and invasive HCC70, when cultured in 3D in the absence or presence of human breast CAFs. Our working hypothesis was that CAFs enhance an invasive phenotype in both preinvasive and invasive BCa cell lines. Over a period of 8 days in TAME 3D culture the BCa cells formed globular structures with smooth borders [Figure 1A,B (MCF10.DCIS) and Figure 2A,B (HCC70)]. Coculture of BCa cells with CAFs resulted in the formation of globular clusters that were larger and more irregular, and exhibited multicellular invasive protrusions (Figure 1C and Figure 2C with corresponding high magnification in Figure 1D and Figure 2D. Red arrows highlight invasive protrusions). There was an increase in volume of the globular structures of both MCF10.DCIS (Figure 1E) and HCC70 cells (Figure 2E), consistent with CAFs inducing an increase in BCa proliferation.

To confirm proliferation versus an increase in cell volume, we quantified total genomic DNA from 8-day TAME 3D cultures. This revealed a significant increase in total DNA present in MCF10.DCIS:CAF cocultures as compared to an equal seeded number of MCF10.DCIS cells grown alone over the same period of time (Supplemental Figure S2). Additionally, the proliferation of CAFs was not significant when grown in TAME 3D monocultures, as shown by total DNA (Supplemental Figure S2A). Furthermore, fluorescent EDU incorporation into DNA provided additional evidence for increased proliferation in our cocultures (Supplemental Figure S2D–F). Previous studies using the 3D coculture of CAFs and MCF10.DCIS cells also showed an increase in DNA synthesis [15]. Thus, we concluded that BCa cells proliferated at an accelerated rate as a result of coculture with CAFs and that CAFs did not significantly contribute to the increased DNA observed in the cocultures.

Our TAME 3D culture model is advantageous in that we can accurately evaluate tumor structure volumes with specificity for volume of cancer cells via differential fluorescent labeling and quantification in 3D of specific cell type(s). Furthermore, minimal physical manipulation of the BCa structure is possible, a feature hard to achieve in animal models due to the need to perform tumor resection. Using confocal microscopy, we evaluated the size, shape, and spatial distribution of RFP-labeled BCa cell structures grown in the absence or presence of unlabeled CAFs. Here, there was a noticeable coalescence of RFP-labeled BCa cells forming large branching structures as a direct result of unlabeled CAFs in the 3D cultures (Supplemental Figure S2E,F).

### 3.2. BCa Cells Represent a Significant Source of Secreted GM-CSF in MCF10.DCIS:CAF Cocultures

**Figure 3:**
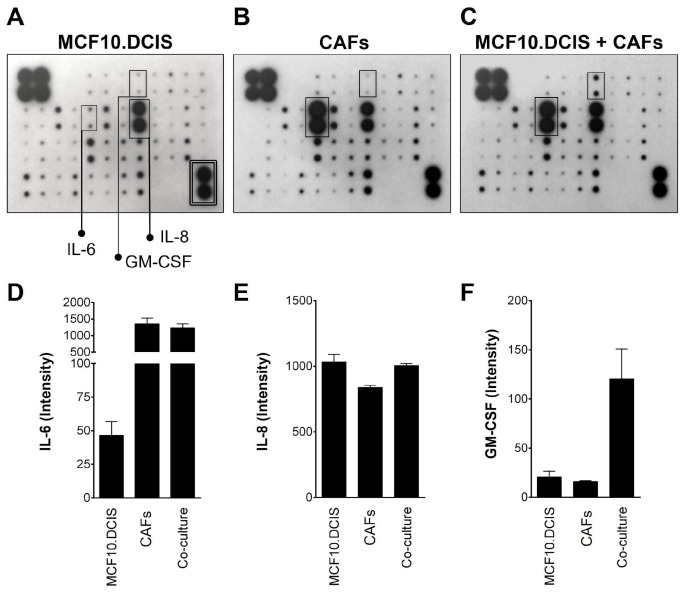
Coculture of MCF10.DCIS cells with CAFs led to an increase in GM-CSF secretion. Scan of inflammation array membrane shows spot intensities of detected cytokines and growth factors. (**A**) MCF10.DCIS cells grown alone in 3D. IL-6, GM-CSF, and IL-8 are boxed and labeled. Positive control double-boxed next to negative controls. (**B**) CAFs grown alone in 3D (IL-6 and GM-CSF boxed). (**C**) 3D coculture of MCF10.DCIS cells and CAFs (IL-6 and GM-CSF boxed). Note the marked induction of GM-CSF in cocultures (**C**), as compared to monocultures (**A**) or (**B**). Spot densitometry quantification of inflammation arrays for the three culture conditions are shown for IL-8 (**D**), IL-6 (**E**) and GM-CSF (**F**). Graphical data are expressed as mean ± standard deviation (*n* = 2).

To identify the secreted factors responsible for the increased BCa cell proliferation and the invasive characteristics of the 3D multicellular structures of MCF10.DCIS:CAF, we utilized an immunoarray targeting known proinflammatory cytokines and growth factors. Conditioned media were collected from 3D cultures of MCF10.DCIS cells alone, CAFs alone, or MCF10.DCIS:CAF cocultures for analysis. We found that the levels of IL-8 were comparable in the CM from all three culture settings (cf. Figure 3A–C, quantified in Figure 3D). Levels of IL-6 were higher in CAF-CM versus MCF10.DCIS-CM (Figure 3A,B,E) and not further elevated in MCF10.DCIS:CAF-CM (Figure 3C,E), consistent with our previous findings that CAFs are the primary source of IL-6 in cocultures [15]. GM-CSF was present at low levels in CM of MCF10.DCIS cells alone (Figure 3A,F) and CAFs alone (Figure 3B,F), yet it was increased in CM of MCF10.DCIS:CAF cocultures (Figure 3C,F). These findings imply a paracrine-mediated induction of GM-CSF due to the interactions between BCa cells and CAFs.

**Figure 4:**
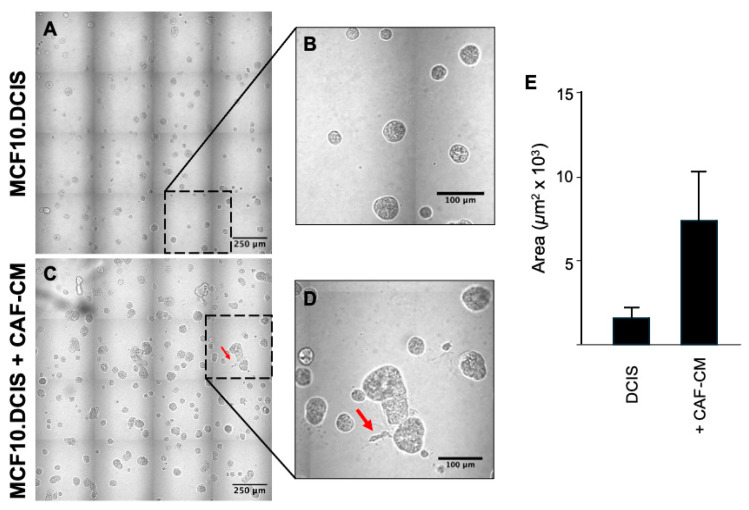
Incubation of BCa cells with CAF-CM resulted in BCa cell proliferation and increased tumor structure volume. MCF10.DCIS cells were grown for a period of 8 days in TAME 3D culture in the absence or presence of CAF-CM. (**A**) Image of 16 contiguous DIC fields of MCF10.DCIS cells in control media or (**C**) in CAF-CM. Note in the high-magnification panels (**B**,**D**) the absence and consequent appearance of protrusions extending from multicellular structures (red arrows). (**E**) Quantification of spheroidal structure area shows a significant increase in BCa treated with CAF-CM. Graphical data are expressed as mean ± standard deviation (*n* = 10). Scale bars: (**A**,**C**) 250 microns; (**B**,**D**) 100 microns.

To evaluate whether soluble factors in CAF-CM could recapitulate the observed changes in BCa cell morphology, we incubated MCF10.DCIS cells with or without CAF-CM for 8 days. Here, we observed similar changes as seen in coculture with CAFs, although to a lesser extent (Figure 4).

### 3.3. CAF-CM Induced GM-CSF Gene Expression via STAT3 in BCa Cells

**Figure 5:**
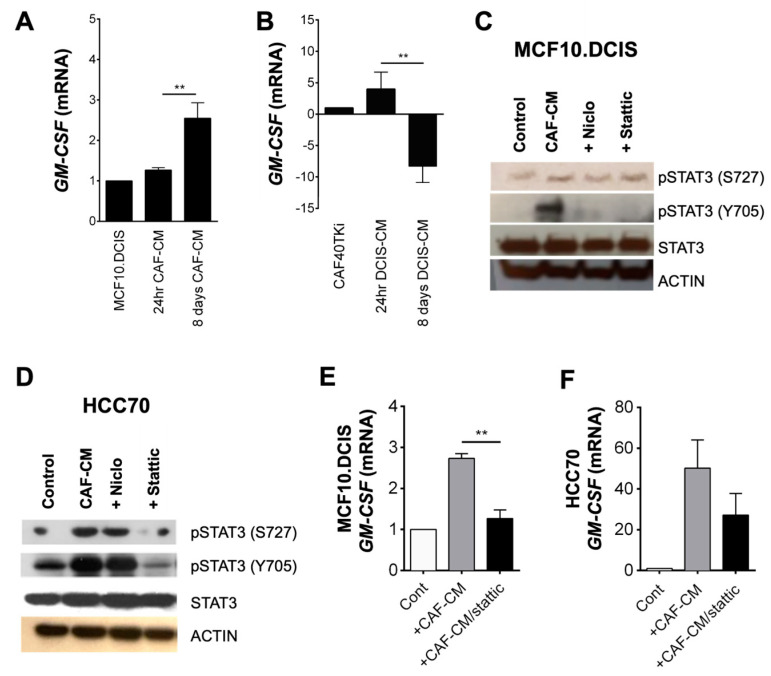
CAF-CM-driven *GM-CSF* expression in cancer cells is mediated by STAT3 activation. (**A**) Immunoblot analysis of lysates from MCF10.DCIS and (**B**) HCC70 cells grown in 2D culture and treated with CAF-CM ± STAT3 inhibitors (niclosamide or stattic). An 8-day 3D culture of MCF10.DCIS cells (**C**) or HCC70 cells (**D**) followed by 24 h exposure to CAF-CM 30 μM static. (**E**,**F**) CAF-CM induced the upregulation of *GM-CSF* in carcinoma cells, which was inhibited by the presence of stattic. The inhibition was statistically significant in the MCF10.DCIS cultures (** *p*-value = 0.01, *n* = 3), but not in HCC70 cultures. Data are expressed as mean ± standard deviation of gene expression fold change using Student’s *t*-test.

To elucidate the source of GM-CSF in our TAME 3D cocultures, we incubated MCF10.DCIS cells with CAF-CM or CAFs with MCF10.DCIS-CM. Evaluation of *GM-CSF* gene expression at 1 day or 8 days after addition of CAF-CM to MCF10.DCIS cells revealed a near-3-fold upregulation of *GM-CSF* expression in MCF10.DCIS cells (Figure 5A). Treatment of CAFs with MCF10.DCIS-CM resulted in an initial 4-fold upregulation of *GM-CSF* at day 1, followed by a greater-than-5-fold downregulation by day 8 (Figure 5B). This inverse gene expression profile suggests the presence of a GM-CSF negative feedback loop in CAFs.

CAF-CM has been shown to drive the expression of mesenchymal markers such as vimentin, twist, and snail1 (all of which are associated with the epithelial-to-mesenchymal transition or EMT) and downregulate epithelial markers like E-cadherin in tumor cells [47,48,49]. STAT3 is a central mediator of EMT transcription factors [50] and is often overexpressed and/or hyper-activated in many cancers including breast cancers [51,52]. Furthermore, phosphorylation of STAT3 at either tyrosine705 (Y705) or serine727 (S727) activates transcriptional expression of various cytokines and growth factors including GM-CSF [53,54]. Thus, it is reasonable to hypothesize that CAF-secreted factors may act in a paracrine manner on neighboring carcinoma cells to activate STAT3, leading to the subsequent expression and secretion of GM-CSF.

To evaluate this hypothesis, we cultured MCF10.DCIS or HCC70 cells with CAF-CM and analyzed lysates for STAT3 phosphorylation. In MCF10.DCIS cultures, CAF-CM induced STAT3 phosphorylation at tyrosine705 in a time-dependent manner (Supplemental Figure S3). Immunoblot analysis also showed an activation of STAT3 in both MCF10.DCIS and HCC70 cells exposed to CAF-CM (Figure 5C,D).

We next evaluated whether blocking CAF-CM-induced STAT3 phosphorylation affected *GM-CSF* gene expression in tumor cells. Here, we used either stattic [55], or niclosamide as selective STAT3 inhibitors. In our study, these cell permeable inhibitors differed in their effects on STAT3 phosphorylation. Niclosamide only slightly inhibited CAF-CM-induced pSTAT3 (S727) in either cell line; however, it near-completely blocked pSTAT3 (Y705) in MCF10.DCIS cells but had little effect in HCC70 cells (Figure 5C,D). In contrast, stattic markedly inhibited CAF-CM-induced pSTAT (S727) in HCC70 cells, but not in MCF10.DCIS cells. Interestingly, stattic blocked the phosphorylation of pSTAT (Y705) in both cell lines. Recombinant IL-6 was used as a positive inducer of STAT3, in which we observed a marked induction of both pSTAT3 (S727) and pSTAT (Y705) (Supplemental Figure S4) [43]. Evaluation of *GM-CSF* gene expression in BCa cells grown in 3D and in the presence of CAF-CM or CAF-CM plus stattic revealed that the STAT3 inhibitor could significantly suppress *GM-CSF* expression in MCF10.DCIS cells (Figure 5E). Stattic blockade of CAF-CM-induced *GM-CSF* gene expression was also observed in HCC70 cells grown in 3D, but this did not meet statistical significance (Figure 5F).

The induction of *GM-CSF* in BCa cells by CAFs or CAF-CM warranted asking how secreted GM-CSF in the TME might affect gene expression in CAFs. To investigate this, we conducted a preliminary gene expression analysis. We treated CAFs with recombinant GM-CSF in 3D culture then extracted RNA to evaluate expression of inflammation-associated genes. We examined *S100A8*, *IL-6*, IL-6R, *IL-8*, and *CXCL3* genes; each of these are known to be differentially expressed in breast cancer and are being evaluated as biomarkers and therapeutic targets [56,57,58,59,60]. We found that each of these genes was upregulated greater than 2-fold in MCF10.DCIS cells treated with CAF-CM or in 3D coculture (Supplemental Table S1). Future studies aim to resolve the signaling networks involved in this GM-CSF-mediated upregulation. In addition, we are examining the roles these inflammation-associated signaling networks play in breast cancer progression.

## 4. Discussion

Exploring the underlying paracrine signaling mechanisms between CAFs and cancer cells will aid in the development of drugs that target paracrine signaling in the TME. CAFs have been shown to drive invasive phenotypes in BCa cells, making CAFs ideal targets for inhibiting tumor progression [61]. In the in vivo tumor setting, paracrine signaling is regulated and propagated by several different cell types, including CAFs. To support our study of paracrine signaling mechanisms between CAFs and BCa cells, we utilized our novel TAME 3D culture model to examine spatiotemporal interactions and paracrine signaling in an in vitro model, which in part mimics the in vivo TME.

We showed that human CAFs induced proliferation of both pre-invasive and invasive human BCa cells, leading to an increased volume of BCa multicellular structures and the formation of invasive processes (Figure 1 and Figure 2). These processes extend out and away from the multicellular mass into the surrounding matrix. Of note is that breast CAFs enhanced the invasive characteristics of the invasive HCC70 cell line. The altered phenotype of multicellular structures in coculture was likely due to both direct cell-to-cell contact and paracrine signaling mechanisms. Secretome analysis data from 3D culture of MCF10.DCIS, CAFs or their cocultures suggested a role for paracrine signaling. The MCF10.DCIS BCa cells and CAFs both secrete a number of cytokines including IL-6, with CAFs secreting almost 30-fold more than MCF10.DCIS cells. These data offer strong evidence for the paracrine signaling-mediated support of tumor growth and progression to an invasive phenotype. However, since we did not observe as dramatic a change in multicellular structures with CAF-CM as compared to the coculture (where cells are in direct contact), we must conclude that a physical cell–cell contact mechanism is involved in promoting an invasive phenotype.

Evaluating the secretomes from MCF10.DCIS or CAF cells vs. the coculture implicated a paracrine activation of GM-CSF as neither cell type secreted equivalent GM-CSF when cultured alone. Gene expression data showing *GM-CSF* expression was upregulated in carcinoma cells exposed to CAF-CM suggest carcinoma cells are the likely source of GM-CSF in coculture. Recent mouse breast cancer model studies suggest that carcinoma cell-derived GM-CSF acts to promote an immunosuppressive TME through paracrine regulation of Arginase-1 in the myeloid cell population, which includes macrophages and fibroblasts [62]. In their study, the researchers showed that BCa-derived GM-CSF lead to a suppressed T-cell function. The paracrine signaling between multiple cell types is part of the next steps in further developing our TAME 3D culture microphysiological system. We plan to coculture in our TAME 3D culture system BCa cells with endothelial cells, fibroblasts, and T-cells to better understand the communication between the different cell types and their coordinated effect on tumor progression.

The treatment of CAFs with MCF10.DCIS-CM yielded an interesting and previously undocumented regulatory mechanism for GM-CSF in CAFs. We observed a brief upregulation in *GM-CSF* gene expression after 24 h exposure followed by a marked decrease by day 8 in 3D culture, thus indicating a strong regulatory process governing *GM-CSF* expression. The molecular mechanisms of this regulation are yet to be uncovered. Studies have shown that *GM-CSF* induction after irradiation can be suppressed by blocking IL-1 signaling [63], which may be a possible mechanism by which *GM-CSF* is downregulated in our system. Other considerations are the involvement of IL-4 [64], IL-10 [65] and interferon gamma [66], of which each has been shown to markedly downregulate *GM-CSF*. Our next steps will include the expansion of our cytokine profiling to evaluate the status of these proteins during the down regulation of *GM-CSF* in our cocultures. Taken together, these data imply that one or more secreted factors present in CAF-CM are responsible for the upregulation of *GM-CSF* in breast cancer cells. Additionally, the downregulation of *GM-CSF* mRNA in CAFs exposed to DCIS-CM suggests a robust negative feedback mechanism, which we will further explore.

STAT3 activation is known to drive the expression of *IL-6*, *TNFα*, *GM-CSF* and many other STAT3 responsive genes [51,67,68]. As CAFs secrete relatively high levels of IL-6, it is likely that IL-6 is responsible for the upregulation of *GM-CSF* mediated through STAT3 activation. To further define the induced *GM-CSF* expression in our cocultures, we evaluated the activation of STAT3 in BCa cells in the presence or absence of CAF-CM and found that CAF-CM activated STAT3 in BCa cells via phosphorylation of both tyrosine 705 [pSTAT3 (Y705)] and serine 727 [pSTAT3 (727)] residues. The addition of the STAT3 inhibitor stattic markedly decreased CAF-CM-induced pSTAT3 (Y705) phosphorylation in both BCa cell lines. Thus, we propose that the secretion of IL-6 from CAFs drives the activation of STAT3 in BCa cells, which in turn drives the expression and secretion of cytokines and growth factors including GM-CSF. The secretion of GM-CSF from tumor cells has also been shown to drive the activation of STAT5 in a subset of macrophages when examined in a breast cancer mouse model [69]. The study further shows that knockout of STAT5 in macrophages cocultured with BCa cells resulted in an increase in tumor cell motility. Here, additional studies are needed to examine how CAFs together with macrophages respond to tumor secreted GM-CSF as CAFs promote tumor survival and invasiveness and while macrophages act in an inhibitory manner. Several research findings indicate that BCa cells exhibit higher expression levels of GM-CSF compared to non-pathogenic cells and tissue [31,70,71,72,73,74]. However, further data are necessary to understand how neighboring cells respond in a coordinated manner to BCa-secreted GM-CSF.

**Figure 6:**
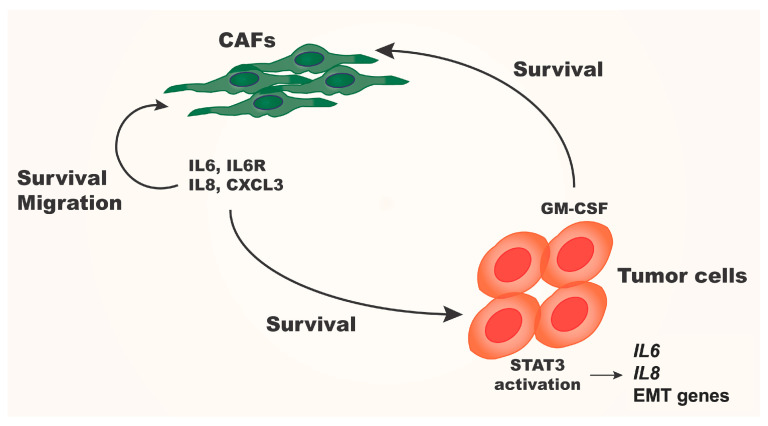
Schematic diagram of pro-tumorigenic paracrine signaling between cancer cells and CAFs. Paracrine cytokines induce STAT3 phosphorylation in cancer cells driving expression of inflammation genes including *IL-6*, IL-8 and *GM-CSF*. Cancer cell-secreted GM-CSF in turn supports fibroblast survival and drives the expression of inflammation-associated cytokines by fibroblasts. Expression and secretion of IL-6, IL-6R, IL-8, and CXCL3 from fibroblasts contributes to paracrine signaling between cancer and non-cancer cells.

In our TAME system, when CAFs were exposed to BCa cell-secreted GM-CSF, they subsequently upregulated the inflammation-associated genes *IL-6*, *IL-6R*, *IL-8* and *CXCL3*, which support both tumor progression and CAF survival. Thus, this formed a positive feedback loop for the continued expression of cytokines and growth factors for both CAFs and cancer cells (Figure 6, Supplemental Table S1). Cho et al. showed that the coculture of human CAFs with human monocytes and treatment with human BCa-CM stimulated CAFs to potentiate monocyte differentiation into M2-like macrophages [35]. This subtype of activated macrophage is known to promote tumor progression, angiogenesis, and tumor drug resistance [75,76]. Our findings are consistent with previous research showing that CAFs support tumor growth. We aim to further our research by investigating the interactions between human BCa cells, endothelial cells, and fibroblasts using our TAME model. This will involve human cells and patient-derived explants, focusing on triple-negative breast cancer due to its high aggressiveness, poor survival rates, and limited effective treatments [77]. By comparing primary and matched metastatic tumor cells in our TAME system, we hope to evaluate the therapeutic efficacy of novel drug candidates on tumor cells at primary and metastatic sites. A recent study has highlighted a potentially targetable difference in gene expression profiles between primary and metastatic tumors [78]. Additionally, our TAME coculture model will provide valuable insights into immune cell behavior and function within the breast cancer tumor microenvironment, offering new immune system targets for the development of immunomodulatory drugs.

## 5. Conclusions

To date, cytokine profiling remains an encouraging method for early detection of breast cancer and for predicting long-term outcomes [64]. However, understanding the pathophysiological functions of secreted cytokines remains a challenging task. Studies aimed at anti-cytokine/growth factor therapies must take into account the cellular source of secreted factors as well as the response of each cell type to the secreted factors. Furthermore, the use of human tissues and human cells in 3D culture models like our TAME system will foster new discoveries and lead to the generation of data that accelerate the translation of basic research to the clinical setting.

## 6. Patents

This publication is associated with the U.S. Patent US10227556B2.

## Data Availability

The data presented in this study are available in this article (and Supplementary Materials).

## Supplementary Materials

The following supporting information can be downloaded at: https://www.mdpi.com/article/10.3390/cancers16162910/s1, Supplemental Figure S1. Diagram of 3D tissue architecture and microenvironment engineering (TAME) cell culture model. Briefly, 100% Cultrex^TM^ was applied to the bottom of cell culture dishes and allowed to solidify in a 5% CO_2_ incubator at 37 °C. CAFs were then added to the Cultrex^TM^ and allowed to adhere for approximately 45 minutes before adding BCa cells at a final ratio of CAFs to carcinoma cells of 1:5. After carcinoma cells adhered, a 2% Cultrex^TM^ overlay was applied. Supplemental Figure S2. Coculture enhanced BCa cell proliferation. (A) Quantification of total genomic DNA from 3D monocultures and cocultures of MCF10.DCIS cells and CAFs. DNA was isolated from both 1-day (open bars) and 8-day (closed bars) cultures. Data were analyzed using Student’s *t*-test (*** *p*-value = 0.001). We observed a marked increase in total DNA content in 3D cocultures as compared to monocultures. (B) DIC image of MCF10.DCIS and CAF cocultures incubated with EDU to evaluate proliferation showed proliferating cells in the invasive protrusions and interconnections between multicellular structures. (C) EDU red fluorescent signal from proliferating cells. (D) Overlay of DIC and red fluorescent signal (scale bars 50 microns). 3D reconstruction of confocal microscopy imaging of RFP-MCF10.DCIS cells grown for 8 days in monoculture (E) and cocultured with unlabeled CAFs (F). BCa cells were labeled with red fluorescent protein and are pseudo colored white. Scale bar equal to 140 microns. Supplemental Figure S3. CAF-CM induced phosphorylation of STAT3 in a time-dependent manner. CAF-CM was added to TAME 3D cultures of MCF10.DCIS cells. Cell lysates were collected at 5, 20, and 30 minutes after addition for analysis of STAT3 (Y705) phosphorylation. CAF-CM activated STAT3 in a time-dependent manner, reaching statistical significance by 30 minutes (*** *p*-value = 0.0004, *n* = 3). Data were quantified using light absorption analysis and are expressed as mean ± standard deviation using one-way ANOVA. Supplemental Figure S4. Densitometry of western blots of STAT3. Densitometry measurements from gels shown in Figure 5C,D. (A–D) MCF10.DCIS cell lines and (E,F) HCC70 cell lines. Measurements were collected using Adobe Photoshop 2024. Supplemental Table S1. Inflammation-associated genes upregulated in CAFs exposed to GM-CSF. CAFs treated with recombinant GM-CSF for 8 days were collected for quantitative mRNA expression analysis of select genes. Employing a significance criterion of 2-fold change or greater, we found an upregulation of each gene we analyzed (*S100A8*, *IL-6*, *IL-6R*, *IL-8*, and *CXCL3*). Data represent average fold change from control ± standard deviation (*n* = 3).
