# Supplementary material for: Paracrine Activation of STAT3 Drives GM-CSF Expression in Breast Carcinoma Cells, Generating a Symbiotic Signaling Network with Breast Carcinoma-Associated Fibroblasts
Source: Cancers (Basel). 2024 Aug 22;16(16):2910. doi: 10.3390/cancers16162910 (PMC11353178; doi:10.3390/cancers16162910)
Supplement: Supplementary file 1 [file cancers-16-02910-s001.zip › cancers-2964844-supplementary.pdf]

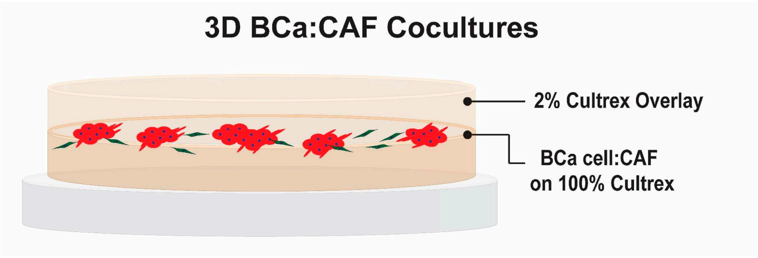

Supplemental Figure S1. Diagram of 3D tissue architecture and microenvironment engineering (TAME) cell culture model. Briefly, 100% Cultrex™ was applied to the bottom of cell culture dishes and allowed to solidify in a 5% CO_2 incubator at 37 °C. CAFs were then added to the Cultrex™ and allowed to adhere for approximately 45 minutes before adding BCa cells at a final ratio of CAFs to carcinoma cells of 1:5. After carcinoma cells adhered, a 2% Cultrex™ overlay was applied.

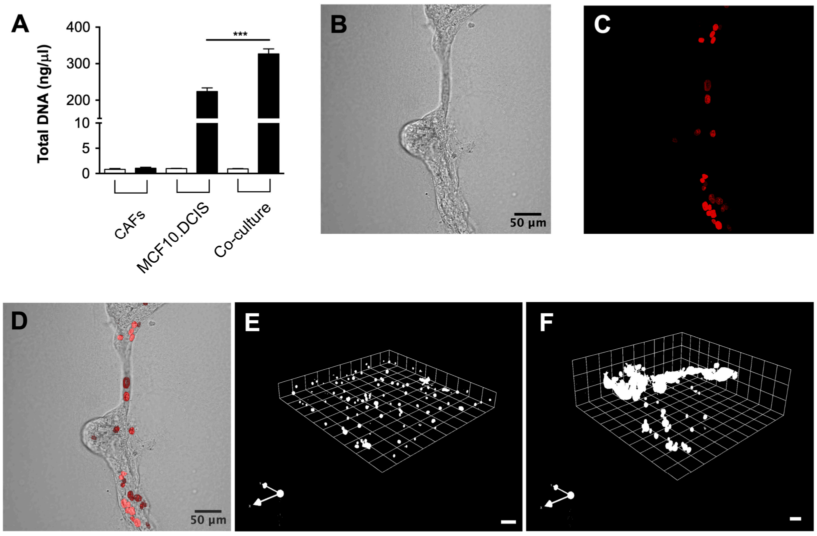

Supplemental Figure S2. Coculture enhanced BCa cell proliferation. (A) Quantification of total genomic DNA from 3D monocultures and cocultures of MCF10.DCIS cells and CAFs. DNA was isolated from both 1-day (open bars) and 8-day (closed bars) cultures. Data were analyzed using Student's t-test (***p-value=0.001). We observed a marked increase in total DNA content in 3D cocultures as compared to monocultures. (B) DIC image of MCF10.DCIS and CAF cocultures incubated with EDU to evaluate proliferation showed proliferating cells in the invasive protrusions and interconnections between multicellular structures. (C) EDU red fluorescent signal from proliferating cells. (D) Overlay of DIC and red fluorescent signal (scale bars, 50 microns). 3D reconstruction of confocal microscopy imaging of RFP-MCF10.DCIS cells grown for 8 days in monoculture (E) and cocultured with unlabeled CAFs (F). BCa cells were labeled with red fluorescent protein and are pseudo colored white. Scale bar equal to 140 microns.

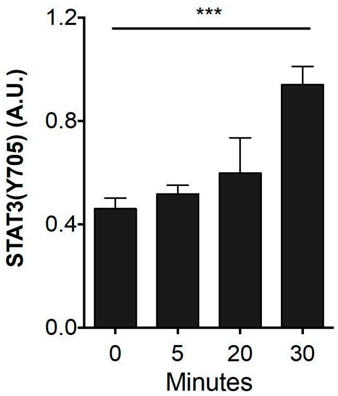

Supplemental Figure S3. CAF-CM induced phosphorylation of STAT3 in a time-dependent manner. CAF-CM was added to TAME 3D cultures of MCF10.DCIS cells. Cell lysates were collected at 5, 20, and 30 minutes after addition for analysis of STAT3 (Y705) phosphorylation. CAF-CM activated STAT3 in a time-dependent manner, reaching statistical significance by 30 minutes (***p-value, 0.0004, *n*=3). Data were quantified using light absorption analysis and are expressed as mean ± standard deviation using one-way ANOVA.

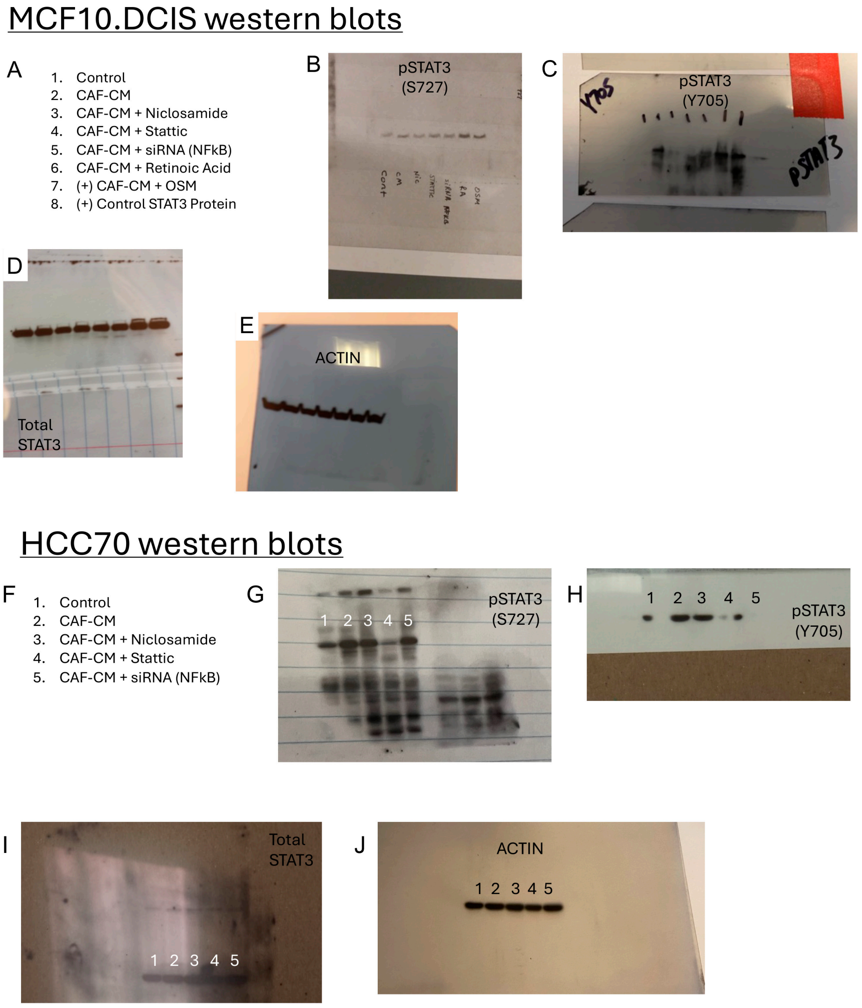

Supplemental Figure S4. Western blots films for MCF10.DCIS (A-E) and HCC70 (F-J). The first four lanes are represented in the main text. All other lanes are not specific to the current study.

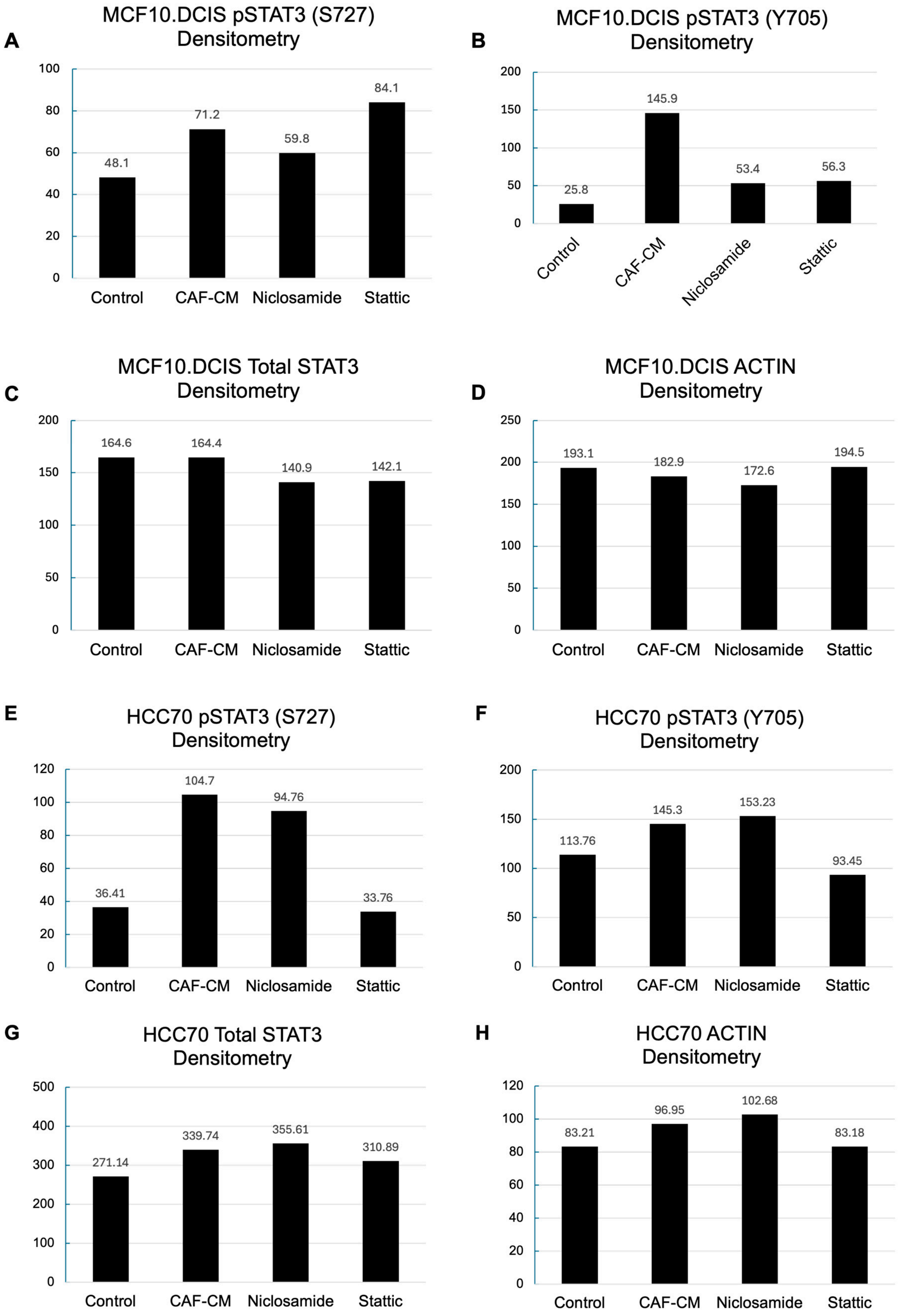

Supplemental Figure S5. Densitometry of western blot lanes 1-4 as shown in main text. Densitometry measurements from gels shown in Figure 5C, D. (A-D) MCF10.DCIS cell lines and (E-F) HCC70 cell lines. Measurements were collected using Adobe Photoshop 2024.

Gene	Fold Change
S100A8	3.7 ± 1.5
IL-6	2.1 ± 0.7
IL-6R	2.9 ± 0.9
IL-8	2.2 ± 0.3
CXCL3	3.0 ± 0.8

Supplemental Table S1. Inflammation-associated genes upregulated in CAFs exposed to GM-CSF. CAFs treated with recombinant GM-CSF for 8 days were collected for quantitative mRNA expression analysis of select genes. Employing a significance criterion of 2-fold change or greater, we found an upregulation of each gene we analyzed (S100A8, IL-6, IL-6R, IL-8, and CXCL3). Data represent average fold change from control ± standard deviation (*n*=3).
